# Attenuated infectious laryngotracheitis virus vaccines differ in their capacity to establish latency in the trigeminal ganglia of specific pathogen free chickens following eye drop inoculation

**DOI:** 10.1371/journal.pone.0213866

**Published:** 2019-03-28

**Authors:** Dulari S. Thilakarathne, Mauricio J. C. Coppo, Carol A. Hartley, Andrés Diaz-Méndez, José A. Quinteros, Omid Fakhri, Paola K. Vaz, Joanne M. Devlin

**Affiliations:** Asia-Pacific Centre for Animal Health, Melbourne Veterinary School, The University of Melbourne, Parkville, Victoria, Australia; Katholieke Universiteit Leuven Rega Institute for Medical Research, BELGIUM

## Abstract

Infectious laryngotracheitis (ILT) is a respiratory disease that affects chickens. It is caused by the alphaherpesvirus, infectious laryngotracheitis virus (ILTV). This virus undergoes lytic replication in the epithelial cells of the trachea and upper respiratory tract (URT) and establishes latent infection in the trigeminal ganglia (TG) and trachea. Live attenuated vaccines are widely used to control ILT. At least one of these vaccines can establish latent infections in chickens, but this has not been demonstrated for all vaccines. The aim of the current study was to determine the capacity of three commercially available vaccines (SA2, A20 and Serva) and a glycoprotein G deletion mutant vaccine candidate (ΔgG ILTV) to establish latent infection in the TG of specific pathogen free (SPF) chickens. Five groups of 7-day-old SPF chickens were eye-drop vaccinated with either one of the vaccine strains or mock-vaccinated with sterile media and followed until 20 or 21 days post-vaccination (dpv). ILTV DNA was detected at 20–21 dpv in the TG of 23/40 (57.5%) vaccinated SPF chickens (SA2 = 10/10; A20 = 6/10; Serva = 3/10; ΔgG = 4/10) by PCR, but virus could not be reactivated from TG co-cultivated with primary chicken embryo kidney cells. In the birds from which ILTV DNA was detected in the TG, ILTV DNA could not be detected in the URT or trachea of 3 birds in each of the SA2, A20 and Serva vaccinated groups, and in 4 birds in the ΔgG vaccinated group, indicating that these birds were latently infected in the absence of active lytic replication and virus shedding. Results from this study demonstrate the capacity of commercial ILTV vaccines to establish latent infections and underline their importance in the epidemiology of this disease.

## Introduction

Infectious laryngotracheitis virus (ILTV) is the causative organism of infectious laryngotracheitis (ILT). ILTV is an alphaherpesvirus that infects the trachea and upper respiratory tract of chickens, causing disease with high morbidity and variable mortality determined by the virulence of the strain involved [1–3]. In the event of an outbreak, ILT causes substantial economic losses associated with drop in egg production [4], weight loss and mortality [1]. Considering the potential risk, especially in areas where the disease is enzootic, intensive poultry producers vaccinate their flocks against ILT [5]. Hitherto, four types of ILTV vaccines have been developed: live vaccines attenuated by sequential passage in embryonated eggs [6] or in tissue culture [7,8], virally vectored recombinant vaccines [9,10] and new generation recombinant deletion mutant vaccines [11–16]. Live attenuated vaccines are the most widely used vaccines worldwide [5]. New generation recombinant deletion mutant vaccines are not yet commercially available. Despite advances in vaccinology, ILTV still represents a significant burden to the poultry industry [17] causing disease in both unvaccinated and vaccinated flocks [18].

Live attenuated ILTV vaccines have several limitations that contribute to the persistence of live viruses in poultry production systems. These viruses multiply within the respiratory tract in birds on administration [19–21] and they are capable of spreading from bird-to-bird within flocks [20–22]. They can revert to virulence after multiple consecutive *in vivo* passages [18], and can recombine and result in the emergence of virulent ILTV strains [23,24]. Importantly, attenuated strains of ILTV also have the potential to establish latent infections in vaccinated birds [25,26].

Latency is a phenomenon observed in all herpesviruses, where much of the knowledge regarding the establishment of latency has been derived from the study of herpes simplex virus 1(HSV-1) [27,28]. During the acute phase of infection, alphaherpesviruses infect the sensory nerve terminals that innervate the primary replication sites and are retrogradely transported to the sensory ganglion where neurone bodies are located. Upon reaching the neuronal nucleus, viral DNA enters the nucleus leaving the capsid behind. The viral DNA genome then becomes associated with histones, is circularised and either undergoes lytic gene expression or suppression, which is thought to be determined by the type of the sensory neurone invaded. Viral genomes that have silenced their lytic gene expression persist within the nucleus as an episome for the life of the host and resume the lytic cycles (reactivation) given the appropriate stimulus [27].

Similar to other alphaherpesviruses, the trigeminal ganglia (TG) has been recognized as the main site of ILTV latency for both vaccine and field strain of virus [21,29–31]. Additionally, Bagust (1986) identified the trachea as a site of ILTV latency by detection of reactivated vaccine viruses in tracheal organ cultures 2, 5, or 10 months post vaccination [32]. Stressors such as changes in housing and onset of sexual maturity act as stimuli for reactivation [26], and these reactivation events either lead to sub-clinical, self-limiting infections or clinical infection that may result in an outbreak of disease [30]. Reactivation of ILTV in vaccinated chickens has been postulated to be an important source of ILTV transmission, especially in long-lived birds such as layers or breeders [33], and may provide a potential source of virus for recombination with other circulating ILTV in a flock [34].

Despite a number of studies showing that attenuated ILTV vaccine strains can be detected in the TG [21,25,29,32], to date no comprehensive comparison has been performed to compare the latency characteristics of different commercially available ILTV vaccines. The aim of this study was to evaluate three commercially available ILTV vaccine strains and a glycoprotein G (gG) deletion mutant ILTV vaccine candidate (ΔgG ILTV) [11] for their ability to establish latent infections in TG of specific pathogen free (SPF) birds following eye-drop inoculation.

## Materials and methods

### Vaccines viruses and cell culture

Three commercial attenuated vaccines MSD Nobilis ILT (MSD, Serva strain), Poulvac Laryngo A20 (Zoetis) and Poulvac Laryngo SA2 (Zoetis) were obtained from the manufacturers for use in this trial. These vaccines were administered at the dose recommended by the manufacturers (≥ 2.5 log_10_ median embryo infective dose (EID_50_), ≥ 10^3.5^ plaque forming units (PFU) and ≥ 10^4.1^ PFU, respectively), as determined by the label information. The ΔgG ILTV vaccine candidate developed by Devlin *et al*. [11] was sourced from our laboratory. This vaccine candidate was propagated and titrated in chicken leghorn male hepatoma (LMH) cells as previously described [11]. This vaccine was administered to birds at a dose of 10^4.0^ PFU per bird, as determined by PFU assay on LMH cells. After administration to the birds, all vaccine strains were re-titrated in a PFU assay using chicken embryo kidney (CEK) cell monolayers, as all the vaccine viruses can be successfully propagated using CEKs. To prepare CEK monolayers, kidneys harvested from the embryos of embryonated SPF eggs at 18 days of incubation were digested with 0.125% w/v trypsin overnight to recover CEK cells. Each well of a twelve-well plate was seeded with approximately 10^5.0^ cells to produce a CEK monolayer as previously described [35].

### *In vivo* study design

This study was conducted with the approval of the Animal Ethics Committee at the Faculty of Veterinary and Agricultural Sciences, The University of Melbourne (Animal Ethics ID: 1513713.1). One hundred SPF hybrid White Leghorn chickens (Australian SPF Services Pty Ltd, Woodend, Australia) were divided into five groups of 20 birds when they were one day old. Each group of birds was housed in separate Horsfall-Bauer type isolators which were equipped with negative pressure HEPA filtered air. Birds received irradiated feed and water *ad libitum*. Wing tags were used for individual bird identification. At seven days of age, all birds in each group were eye-drop vaccinated with one dose of one of the four ILTV vaccines; Serva, A20, SA2, or ΔgG ILTV, or were mock inoculated with sterile cell culture medium (Dulbecco’s Modified Eagle’s Medium [DMEM] supplemented with 10% v/v foetal bovine serum [FBS]). In all groups, the volume of the inoculum was 30 μl, which was administered to the left eye of each bird. Birds were monitored for up to 21 days post vaccination (dpv) for clinical signs of ILT.

### Sample collection

Tracheal swabs were collected from all birds 4 and 14 dpv. On 20 and 21 dpv, 10 birds in each group were euthanised by halothane exposure and a detailed post-mortem examination was conducted on each bird. Swabs were collected from the conjunctival mucosa, palatine cleft, infraorbital sinus and trachea of each bird, to test for the presence of vaccine virus in primary replication sites. All samples were collected into 1 ml of sterile viral transport media consisting of DMEM supplemented with 3% v/v FBS, 0.02 M 4-(2-hydroxyethyl)-1-piperazineethanesulfonic acid (HEPES, pH 7.7) and 0.25 mg/ml of each gentamicin and ampicillin, and stored at -80°C until further processing.

On the day of necropsy, swabs (conjunctival mucosa, palatine cleft, infraorbital sinus and trachea) were taken from all birds prior to dissection. Separate instruments and workspaces were used for opening the birds and the removal of TG. An entirely new set of instruments was used for each vaccine group. Instruments were cleaned and sterilised by application of 70% (v/v) ethanol and flaming between birds within a vaccine group. Workspaces were washed down after applying sodium hypochlorite solution and cleaned with 80% ethanol between vaccinated groups. Left and right TG collected from each bird 20 dpv were minced and divided into two fractions each and stored in 0.5 ml of sterile viral transport media (composition as above). One fraction was kept in wet ice and used for immediate TG co-culture (see below). The other fraction was immediately frozen in dry ice until permanent storage at -80°C. Left and right TG collected from each bird on 21dpv were pooled and stored in 0.5 ml of sterile viral transport media at -80°C until they were used for molecular detection of ILTV by polymerase chain reaction (PCR).

### Co-culture of trigeminal ganglia

Co-culture of TG was performed according to the method described by Mostafa *et al*. [36] with modifications. Each TG fraction transported in wet ice on 20 dpv was added to a well in a twelve-well plate, containing a sub confluent (~80–90%) CEK monolayer and 1 ml of growth medium (DMEM supplemented with 5% v/v FBS, 2 μM L-glutamine, 2.5 μg/ml amphotericin B and 0.2 mg/ml of each ampicillin and gentamicin. Co-cultures were incubated at 37°C in a humidified atmosphere with 5% (v/v) CO_2_. Five hundred microliters of culture supernatants were collected daily and stored at -80°C for subsequent testing for the presence of reactivated ILTV, and the same volume was replaced with fresh media. Depending on the integrity and viability of the CEK monolayer, TG tissues were transferred to a new monolayer every 5^th^ day and co-cultures were maintained for up to 15 days. Supernatants were analysed for the presence of ILTV DNA using nested PCR. Cultures negative for ILTV DNA, even at 10^th^ day post-explant, were given a heat shock at 43°C for 3 hours before re-plating to provide an added stimulus for viral reactivation [36].

### Molecular detection of ILTV DNA in swabs using UL15 qPCR

DNA was extracted from 200 μl of swab supernatants of conjunctival mucosa, palatine cleft, infraorbital sinus and tracheal swabs, or culture supernatants, using the PureLink Pro 96 viral RNA/DNA purification kit (Invitrogen, Carlsbad, California, United States) and the Corbett X-tractor Gene Robot (Corbett Life Science Pty Ltd, Sydney, Australia). For DNA extraction from TG collected on 21 dpv, Roche High Pure PCR template preparation kit (Roche diagnostics GmbH, Mannheim, Germany) was used according to manufacturer's instructions and DNA was eluted in 50 μl of elution buffer.

Quantitative polymerase chain reaction (qPCR) assay was used to quantify ILTV genome copy number (GCN) in all collected swab samples. Extracted DNA was subjected to qPCR targeting a 113 bp fragment in the UL15 gene of the ILTV genome, following the protocol by Mahmoudian *et al*. [37]. As this qPCR is convenient to apply to large numbers of samples, all collected swab samples were first screened for quantifiable ILTV DNA using this PCR. Selected negative swab samples were then further tested to qualitatively detect ILTV DNA using a more sensitive nested PCR which is more resource intensive to perform on a large scale.

### Molecular detection of ILTV DNA in TG tissue and selected swabs using nested PCR

A nested PCR was used to test for the presence of ILTV DNA in TG collected on 21 dpv, TG co-culture supernatants and selected swabs collected on 21 dpv. Conjunctival mucosa, palatine cleft, infraorbital sinus and tracheal swabs were only tested for the presence of ILTV DNA using the nested PCR if the swabs collected 21 dpv tested negative for ILTV DNA using the less sensitive UL15 qPCR, and if the TG sample from the same individual bird was positive for ILTV using the nested PCR. This nested PCR, the universal herpes virus (UHV) nested PCR, targets a conserved region of the herpes virus DNA polymerase gene [38]. Products amplified during the second round of this PCR were purified using QIAquick Gel Extraction Kit (QIAGEN, Hilden, Germany) following the manufacturer’s instructions. Depending on the yield of purified DNA, amplicons were either directly subjected to sequencing or cloned into pGEM-T (Promega) following manufacturer’s instructions before sequencing. Sequencing reactions were performed using Big Dye Terminator v3.1 (Life Technologies Corporation, Carlsbad, USA) according to manufacturer’s instructions. Sample electrophoresis and sequencing was performed at the Centre for Translational Pathology, University of Melbourne. Geneious software version 9.1.3 [39] was used to analyse the sequencing data.

### Statistical analysis

Microsoft Excel was used for log transformation of genome copy number (GCN) and Minitab statistical software version 18 (Minitab Pty Ltd, Sydney, Australia) was used to perform statistical analyses. Comparison of viral GCN was performed using one-way analysis of variance in conjunction with Dunnett’s multiple comparison test. Comparisons of the proportion of ILTV DNA positives between groups was done using Fisher’s exact test. *P* values ≤ 0.05 were considered statistically significant.

## Results

### *In vivo* study

None of the birds showed any clinical signs of ILT after vaccination. Re-titration of inoculum in CEK monolayers produced titres of 10 ^3.70^, 10 ^3.94^, 10 ^5.08^, and 10 ^6.58^ PFU/ml for Serva, A20, SA2 and ΔgG ILTV, respectively.

### Detection of ILTV DNA in swab samples using UL15 qPCR

The presence of ILTV DNA in each swab sample collected per time point, was quantified using UL15 qPCR. Samples containing GCN of ≥ 200 per reaction were considered positive. The results from these assays are presented in Table 1. The highest proportion of positive swabs was detected at either 14 dpv (Serva) or day 4 dpv (all other vaccines). At 4 dpv, a significantly higher proportion of positive tracheal swabs were detected in birds vaccinated with SA2 (20/20) and A20 (18/20), compared to the other groups. At 4 dpv, the mean GCN in the SA2 and A20 vaccinated groups were significantly higher than the GCN in the groups of chickens vaccinated with Serva or ΔgG ILTV (P < 0.001), and the SA2 vaccinated group GCN were significantly higher than the A20 group (*P* < 0.001). At 14 dpv a significantly higher proportion of positive tracheal swabs were detected in birds vaccinated with Serva (5/20) compared to the other groups. There was no significant difference at 14 dpv in the GCN, between the different groups of birds.

**Table 1:** Proportion of ILTV positive swabs and genome copy numbers in samples collected from SPF chickens after eye-drop vaccination with Serva, A20, SA2, ΔgG ILTV, or mock inoculation with sterile media.

Group	4 dpv^‡^		14 dpv		20 or 21 dpv							
	Trachea		Trachea		Conjunctiva		Palatine cleft		Infraorbital sinus		Trachea	
	Proportion of positives	Mean Log_10_ GCN^¶^ ± SD Log_10_^#^	Proportion of positives	Mean Log_10_ GCN ± SD Log_10_	Proportion of positives	Mean Log_10_ GCN ± SD Log_10_	Proportion of positives	Mean Log_10_ GCN ± SD Log_10_	Proportion of positives	Mean Log_10_ GCN ± SD Log_10_	Proportion of positives	Mean Log_10_ GCN ± SD Log_10_
**Control**	(0/20) ^a^	2.30 ± 0.00^§^ ^a^	(0/20) ^a^	2.30 ± 0.00 ^a^	(0/20) ^a^	2.30 ± 0.00 ^a^	(0/20) ^a^	2.30 ± 0.00 ^a^	(0/20) ^a^	2.30 ± 0.00 ^a^	(0/20) ^a^	2.30 ± 0.00 ^a^
**Serva ILTV**	(2/20) ^a^	2.36 ± 0.02 ^a^	(5/20) ^b^	2.50 ± 0.57 ^a^	(1/20) ^a^	2.36 ± 0.25 ^a^	(3/20) ^a^^,^ ^d^	2.36 ± 0.15 ^a^	(0/20 ^a^	2.30 ± 0.00 ^a^	(1/20) ^a^	2.31 ± 0.03 ^a^
**A20 ILTV**	(18/20) ^b^	3.85 ± 0.95 ^b^	(1/20) ^a^	2.31 ± 0.05 ^a^	(2/20) ^a^	2.40 ± 0.30 ^a^	(5/20) ^b^^,^ ^d^	2.35 ± 0.13 ^a^	(1/20) ^a^	2.31 ± 0.02 ^a^	(2/20) ^a^	2.35 ± 0.21 ^a^
**SA2 ILTV**	(20/20) ^b^	5.16 ± 0.34 ^c^	(2/20) ^a^	2.32 ± 0.06 ^a^	(1/20) ^a^	2.33 ± 0.12 ^a^	(11/20) ^b^^,^ ^e^	2.53 ± 0.30 ^b^	(2/20) ^a^	2.36 ± 0.23 ^a^	(5/20) ^b^	2.40 ± 0.18 ^a^
**ΔgG ILTV**	(2/20) ^a^	2.37 ± 0.25 ^a^	(0/20) ^a^	2.30 ± 0.00 ^a^	(0/20) ^a^	2.30 ± 0.00 ^a^	(0/20) ^a^	2.30 ± 0.00 ^a^	(0/20) ^a^	2.30 ± 0.00 ^a^	(0/20) ^a^	2.30 ± 0.00 ^a^

^**a, b, c, d, e**^ Values marked with a different lowercase superscript character in each column were statistically significantly different (p < 0.05)

^**§**^ The limit of detection using this qPCR was log_10_ (2.3) GCN per reaction. All samples that returned GCN values at or below this limit were determined to be negative and were assigned this GCN value for use in subsequent statistical analyses

^**#**^ SD: Standard deviation

^‡^ dpv: Days post vaccination

^¶^ GCN: Genome copy number

At 20 or 21 dpv, the swabs collected from the palatine cleft accounted for the highest proportion of samples positive for ILTV DNA. Birds vaccinated with SA2 had the highest proportion of UL15 qPCR positive palatine cleft swabs (11/20), compared to all other vaccinated groups (*P* = 0.001). The ILTV GCN detected in SA2 vaccinated birds was also significantly higher than that observed in the other groups of vaccinated birds in this same anatomical site. The remaining sites of the URT that were tested for the presence of ILTV DNA yielded low proportions of positive samples. The only significant difference found at these other sites was a higher proportion of ILTV DNA positive tracheal swabs of SA2 vaccinated birds (5/20) compared to all other groups (*P* = 0.047). ILTV DNA was not detected at this time point in any of the mock-vaccinated chickens or in those vaccinated with ΔgG ILTV.

### Detection of ILTV DNA in TG tissue, TG co-cultures and selected swab samples using UHV nested PCR

The TG collected during post-mortem examination were subjected to two different techniques for viral detection. Half of the birds in each group (n = 10) were culled on 20 dpv and their TG were co-cultured in CEK monolayers. The remaining birds (n = 10) were culled 21 dpv, and their TG were used in the molecular detection of ILTV genomes using the UHV nested PCR. ILTV DNA was detected in TG tissue using UHV nested PCR in 3/10 TG collected from the Serva vaccinated group, 4/10 TG from the ΔgG ILTV vaccinated group, 6/10 TG from the A20 vaccinated group and 10/10 TG from the SA2 vaccinated group (Table 2 and S1 Table). There was a significantly higher proportion of ILTV in the TG of the SA2 vaccinated group, compared with the Serva and ΔgG ILTV vaccinated groups (*P* ≤ 0.01, Fisher’s exact test). Sanger-sequencing confirmed the presence of ILTV DNA in 14/23 of the TG positive samples, all of which were collected from birds inoculated with SA2 or A20 ILTV.

**Table 2:** Detection of ILTV DNA by quantitative and nested PCRs in swabs collected from the upper respiratory tract or trachea of SPF chickens after vaccination with Serva, SA2, A20 or ΔgG ILTV. Only results from birds that had detectable levels of ILTV DNA in their trigeminal ganglia are shown.

Group/Bird ID	UL15 qPCR Log_10_*						UHV Nested PCR^				
	4 dpv^‡^ Trachea	14 dpv Trachea	21 dpv Eye	21 dpv Infraorbital sinus	21 dpv Palatine cleft	21 dpv Trachea	21 dpv Eye	21 dpv Infraorbital sinus	21 dpv Palatine cleft	21 dpv Trachea	21 dpv TG
Serva_NO^†^_3	-	2.48	-	-	-	-	-	-	-	-	+
Serva_7233	2.75	-	-	-	-	-	-	-	-	-	+
Serva_7235	-	-	-	-	-	-	-	-	-	-	+
A20_7247	2.76	-	-	-	-	3.23	NT ^Ʃ^	NT	NT	NT	+
A20_NO_2	3.84	-	-	-	-	-	-	-	-	-	+
A20_7252	4.59	-	-	-	-	-	-	-	-	-	+
A20_7256	3.88	-	-	-	-	-	-	-	-	-	+
A20_7257	3.26	-	-	-	-	-	-	+	+	-	+
A20_7260	4.16	-	-	-	-	-	-	-	+	-	+
SA2_7262	5.10	-	-	-	2.65	2.79	NT	NT	NT	NT	+
SA2_7263	5.09	2.42	-	-	3.33	-	NT	NT	NT	NT	+
SA2_7264	5.03	-	-	-	2.40	-	NT	NT	NT	NT	+
SA2_7265	5.12	-	-	-	-	-	-	-	-	-	+
SA2_7266	5.30	-	-	-	2.85	-	NT	NT	NT	NT	+
SA2_7267	4.91	-	-	3.33	3.11	2.44	NT	NT	NT	NT	+
SA2_7272	4.71	-	-	-	-	-	-	-	-	-	+
SA2_7274	4.48	-	-	2.50	2.64	-	NT	NT	NT	NT	+
SA2_7275	5.17	-	-	-	-	-	-	-	-	-	+
SA2_7280	5.11	-	-	-	2.60	2.66	NT	NT	NT	NT	+
ΔgG_7281	-	-	-	-	-	-	-	-	-	-	+
ΔgG_7282	-	-	-	-	-	-	-	-	-	-	+
ΔgG_7289	-	-	-	-	-	-	-	-	-	-	+
ΔgG_7293	-	-	-	-	-	-	-	-	-	-	+

^ The UHV nested PCR was applied to swabs collected 21 dpv only if the UL15 qPCR did not detect ILTV DNA in any of the swabs collected 21 dpv, and only if the TG tissue was positive for ILTV DNA

^‡^ dpv: Days post vaccination

^†^ NO: Bird lost its identification tag during the trial

^Ʃ^ NT: Not tested with NPCR

* Copies of ILTV DNA per reaction

**Fig 1:**
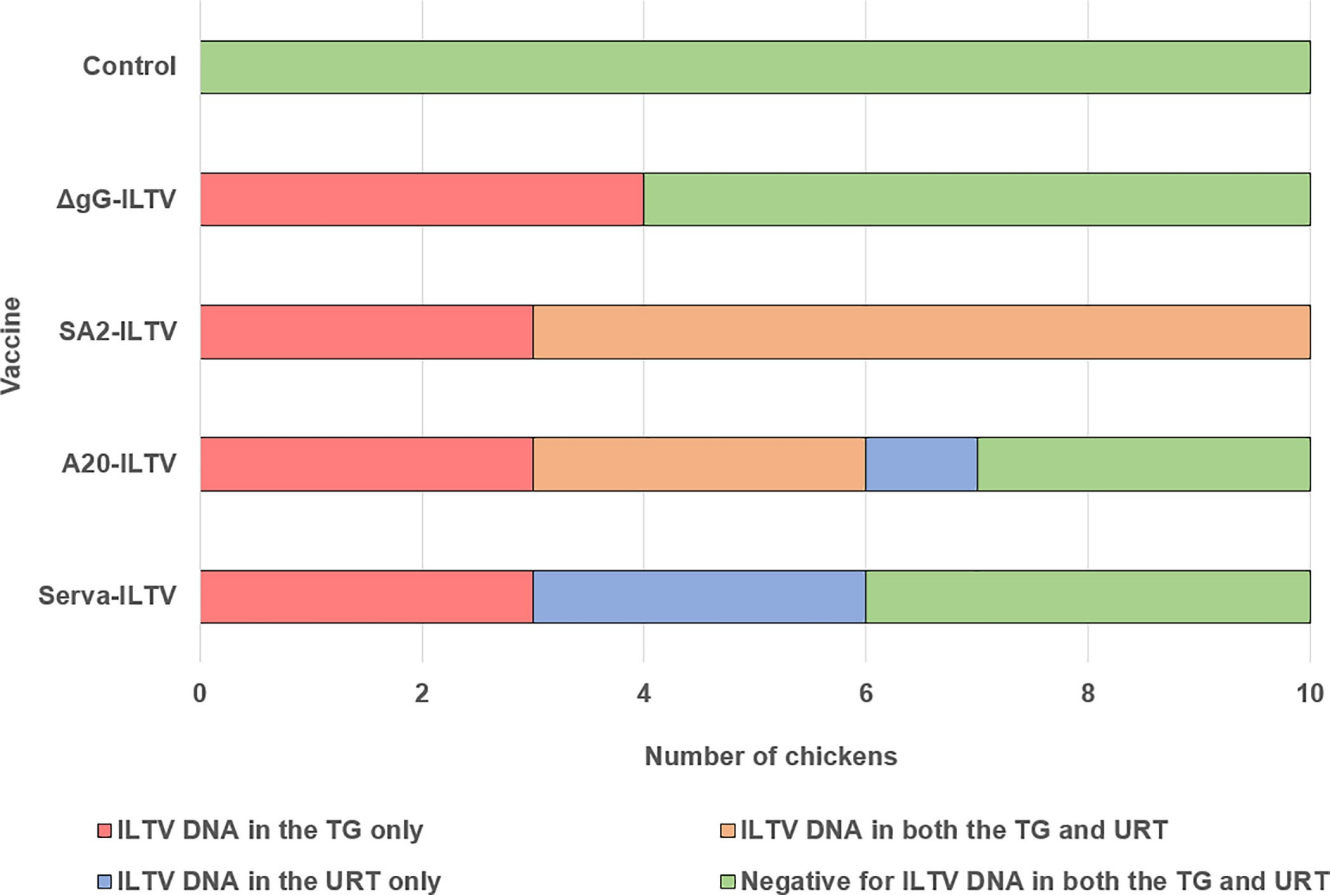
Summary of ILTV DNA detection results at day 21 post vaccination. The presence of ILTV DNA in the TG only suggests latent infection. The presence of ILTV DNA in the TG and URT suggests concurrent latent and lytic infection. The presence of ILTV DNA in the URT only suggests lytic infection.

In those individual birds where ILTV DNA was detected in the TG tissue using UHV nested PCR, but not in swabs collected at 21 dpv using UL15 qPCR, the swabs were re-tested with the UHV nested PCR. This was performed to identify birds that were actively shedding virus at these sites. A total of 3 birds in each of the Serva, A20, and SA2 groups, and 4 birds in the ΔgG ILTV group (Table 2 and supporting information S1 Table) had ILTV DNA in the TG without detectable virus shedding at any other sites and therefore were classifid as being latently infected without any concurrent lytic infection. Swab samples collected from birds in the mock-vaccinated group were tested in parallel with the other groups and all of them were negative for ILTV DNA. A summary of ILTV DNA detection results in TG and swabs using PCR at 21 dpv is presented in Fig 1. TG co-culture supernatants were tested up to 16 days post explantation using the UHV nested PCR, but viral DNA was not detected in any of the samples.

## Discussion

In this study, four ILTV vaccine strains were analysed for their ability to establish latent infection in chickens following eye-drop administration. The duration and sampling time points used in the current study were based on previous ILTV studies [1,20,40] and were selected to capture the replication kinetics of the vaccine strains. While vaccine doses used in this study were those defined by the manufacturer or similar, re-titration of these doses in CEK cells showed doses for the different vaccines ranged from 10 ^3.70^ (Serva) to 10 ^6.58^ (ΔgG) PFU/ml.

Tracheal swabs were collected on 4 dpv and 14 dpv to identify active viral replication and cessation of viral replication, respectively. There was a high proportion of ILTV-positive tracheal swabs in the SA2 and A20 vaccinated groups early after vaccination, which then decreased at later time points and a lower proportion of ILTV positive birds in the Serva and ΔgG vaccinated groups were detected early after vaccination, compared with the other vaccinated groups. These results are largely consistent with previous studies [11,20,41], but the results from the Serva vaccinated group 4 dpv are in contrast to findings by Coppo *et al*. [19]. In Coppo *et al*.[19] study, a high proportion (1.0) of UL15 qPCR positive birds were detected 4 dpv following vaccination with a similar dose of Serva and using the same route of administration. Future studies may require the collection of samples from different anatomical sites (i.e. conjunctival swabs, palatine cleft swabs) at 4 dpv in order to better understand the replication characteristics of this virus after eye-drop vaccination.

Generally acute ILTV infections subside within 14 days post-infection and therefore final samples were collected at 20 or 21 dpv, as this is after the expected cessation of viral replication from the initial inoculation of virus. At the final time point, 26/80 (32.5%) of the vaccinated birds had viral DNA in at least one of the sites from which swab samples were collected (i.e. conjunctiva, palatine cleft, infraorbital sinus and trachea). This observation is consistent with a previous study that detected a high proportion of UL15 qPCR positives in tracheal swabs collected 21 dpv with the same ILTV strains, administered via the same route [20]. The high proportion of ILTV-positive birds in the SA2 vaccinated group is also consistent with previous results showing a high proportion (15/19) of ILTV DNA positive tracheas in chickens 21 dpv with SA2 [20]. Whether this DNA is coming from infectious viruses that are shed as a result of reactivation of latent viruses, re-infection of the respiratory tract with virus shed from other birds, or whether this DNA is remnant DNA from vaccine viral replication after inoculation is currently unknown.

Latent ILTV could not be detected using the TG co-culture system implemented for this study. There are several possible explanations for this outcome, including: the division of the TG in half before co-culture (performed so that TG tissue could be directed towards different analytical methods), a potentially low number of neurones being infected after only one infection event, and dilution of the virus in the supernatant due to the sampling technique used at each time point during co-culture. In murine models of HSV-1 infection, approximately 5% of the neurones are latently infected [42] and among them, only a small number (1 in 2700 latently infected cells) have the capability of reactivation *in vivo* [43]. A similar phenomenon may occur during ILTV infection and future studies to examine ILTV latency may have to consider these limitations in the study design.

Viral DNA was detected in the TG tissue collected from 23/40 (57.5%) vaccinated birds 21 dpv. ILTV DNA was not detected anywhere else in 13 of these birds when tested using the UHV nested PCR. It is likely that these 13 birds represent latently infected animals without any concurrent lytic replication of virus occurring at other sites. Ten other birds had detectable ILTV DNA in TG but were also ILTV-positive in the URT or trachea. It is also possible that these birds may represent birds with latent infections that have been reactivated, as 9/10 of these birds showed cessation of viral active viral replication in trachea by 14 dpv, however re-infection with virus excreted from other birds in the group may also be possible. Although the trachea has been identified as a site of ILTV latency [32] it is unlikely that our approach of swabbing the trachea was able to detect latent virus at this site. The tracheal neurons are typically located in the adventitia and our swab sampling method only reaches the tracheal mucosa. It is also possible that chickens that have latent infections in their TG may not have latent infections in the trachea, and vice versa. It is likely that the route of inoculation and the site of primary replication play a role in the establishment of latent infections at each site.

Despite receiving a comparable dose of virus, viral DNA was detected in a higher proportion of birds vaccinated with SA2 (100%), compared to those vaccinated with ΔgG ILTV (40%). It is interesting to note that virus was detected in the TG of a small proportion of ΔgG ILTV vaccinated birds (not significantly different to the mock-vaccinated group) and also in the swab samples of a small proportion of ΔgG ILTV vaccinated birds. In contrast, SA2 was detected in the TG of all vaccinated birds and in the swab samples of most SA2 vaccinated birds. It is possible that a higher level of virulence in SA2 [1,32] is associated with greater viral persistence in the trachea and URT, and enhanced infection of sensory neurones and uptake by the TG, and vice versa for the ΔgG ILTV vaccine candidate. Studies of HSV-1 have shown that highly attenuated deletion mutants deficient in one or more critical genes are still capable of establishing latent infections [44–53]. Further, wild-type HSV-1 has been reported to establish latency in sensory ganglia that do not innervate the primary replication sites [54]. It would be interesting to investigate the association between ILTV strain-specific virulence and their potential to establish latency.

Nested PCR was used in the current study to detect viral DNA. This technique has been an important molecular tool for the detection of ILTV DNA in TG due to its high level of sensitivity [25,55–57]. However, detection of latency using PCR-based methods has two limitations: i) inability to differentiate between lytic and latent genomes and ii) incapability of predicting reactivation potential of the detected viruses in sites of latency. In murine models of HSV-1 infection, occasional viral transcriptional activation has been seen in a small number of latently infected neurones while the remaining majority are silent, resulting in molecular reactivations without clinical disease or viral shedding [58]. Ideally, approaches to better assess ILTV latent infections in TG would include a combination of TG explantation and co-culture together with in-situ RT-PCR targeting latency associated transcripts (LATs). However, LATs associated with ILTV latency remain poorly understood [59,60] and *in vitro* TG explant reactivation systems are yet to be optimised.

This study has revisited an aspect of the pathogenesis of ILTV that has received little attention in recent years. The results from this study have confirmed results from early studies and have also added new information to our understanding of ILTV vaccine latency. The finding that attenuated ILTV vaccines differ in their capacity to establish latency may have practical relevance for control of ILTV in the field, as selecting vaccines that have a limited ability to establish latency may help to reduce the number of disease outbreaks that result from reactivation of latent vaccine virus. Future studies to establish consistent and reliable latency models of ILTV in the natural host and *in vitro* models of reactivation are warranted in prospects of future understanding of the pathogenesis of ILTV.

## Supporting information

S1 Table Detection of ILTV DNA by quantitative and nested PCRs in swabs collected from upper respiratory tract, trachea or trigeminal ganglia (TG) of SPF chickens at day 21 after eye-drop vaccination with Serva, SA2, A20, ΔgG ILTV or sterile media (control). (DOCX)
