# Supplementary material for: Attenuated infectious laryngotracheitis virus vaccines differ in their capacity to establish latency in the trigeminal ganglia of specific pathogen free chickens following eye drop inoculation
Source: PLoS One. 2019 Mar 28;14(3):e0213866. doi: 10.1371/journal.pone.0213866 (PMC6438565; doi:10.1371/journal.pone.0213866)
Supplement: S1 Table — (DOCX) [file pone.0213866.s001.docx]

**S1 Table:** Detection of ILTV DNA by quantitative and nested PCRs in swabs collected from upper respiratory tract, trachea or trigeminal ganglia (TG) of SPF chickens at day 21 after eye-drop vaccination with Serva, SA2, A20, ΔgG ILTV or sterile media (control).

| Group/  Bird ID | **UL15 qPCR**  **Log_10_** | | | |  | **Nested PCR^** | | | | | | |
| --- | --- | --- | --- | --- | --- | --- | --- | --- | --- | --- | --- | --- |
|  | Eye | Infraorbital sinus | Palatine cleft | Trachea |  | Eye | Infraorbital sinus | | | Palatine cleft | Trachea | TG |
| Control_7202 | - | - | - | - |  | NT^Ʃ^ | | NT | NT | | NT | - |
| Control_7203 | - | - | - | - |  | NT | | NT | NT | | NT | - |
| Control_NO^†^_1 | - | - | - | - |  | NT | | NT | NT | | NT | - |
| Control_7206 | - | - | - | - |  | NT | | NT | NT | | NT | - |
| Control_7208 | - | - | - | - |  | NT | | NT | NT | | NT | - |
| Control_7210 | - | - | - | - |  | NT | | NT | NT | | NT | - |
| Control_7211 | - | - | - | - |  | NT | | NT | NT | | NT | - |
| Control_NO_2 | - | - | - | - |  | NT | | NT | NT | | NT | - |
| Control_7215 | - | - | - | - |  | NT | | NT | NT | | NT | - |
| Control_7218 | - | - | - | - |  | NT | | NT | NT | | NT | - |
| Serva_NO_1 | - | - | 2.75^*^ | - |  | NT | | NT | NT | | NT | - |
| Serva_7224 | - | - | - | - |  | NT | | NT | NT | | NT | - |
| Serva_7225 | - | - | 2.54 | - |  | NT | | NT | NT | | NT | - |
| Serva_7226 | - | - | - | - |  | NT | | NT | NT | | NT | - |
| Serva_7228 | - | - | - | - |  | NT | | NT | NT | | NT | - |
| Serva_NO_2 | - | - | - | - |  | NT | | NT | NT | | NT | - |
| Serva_NO_3 | - | - | - | - |  | - | | - | - | | - | + |
| Serva_7233 | - | - | - | - |  | - | | - | - | | - | + |
| Serva_7235 | - | - | - | - |  | - | | - | - | | - | + |
| Serva_7238 | - | - | 2.82 | - |  | NT | | NT | NT | | NT | - |
| A20_7244 | - | - | - | - |  | NT | | NT | NT | | NT | - |
| A20_NO_1 | - | - | - | - |  | NT | | NT | NT | | NT | - |
| A20_7247 | - | - | - | 3.23 |  | NT | | NT | NT | | NT | + |
| A20_NO_2 | - | - | - | - |  | - | | - | - | | - | + |
| A20_NO_3 | - | - | - | - |  | NT | | NT | NT | | NT | - |
| A20_7252 | - | - | - | - |  | - | | - | - | | - | + |
| A20_7256 | - | - | - | - |  | - | | - | - | | - | + |
| A20_7257 | - | - | - | - |  | - | | + | + | | - | + |
| A20_7258 | 3.31 | - | - | 2.40 |  | NT | | NT | NT | | NT | - |
| A20_7260 | - | - | - | - |  | - | | - | + | | - | + |
| SA2_7262 | - | - | 2.65 | 2.79 |  | NT | | NT | NT | | NT | + |
| SA2_7263 | - | - | 3.33 | - |  | NT | | NT | NT | | NT | + |
| SA2_7264 | - | - | 2.40 | - |  | NT | | NT | NT | | NT | + |
| SA2_7265 | - | - | - | - |  | - | | - | - | | - | + |
| SA2_7266 | - | - | 2.85 | - |  | NT | | NT | NT | | NT | + |
| SA2_7267 | - | 3.33 | 3.11 | 2.44 |  | NT | | NT | NT | | NT | + |
| SA2_7272 | - | - | - | - |  | - | | - | - | | - | + |
| SA2_7274 | - | 2.50 | 2.64 | - |  | NT | | NT | NT | | NT | + |
| SA2_7275 | - | - | - | - |  | - | | - | - | | - | + |
| SA2_7280 | - | - | 2.60 | 2.66 |  | NT | | NT | NT | | NT | + |
| ΔgG_7281 | - | - | - | - |  | - | | - | - | | - | + |
| ΔgG_7282 | - | - | - | - |  | - | | - | - | | - | + |
| ΔgG_7283 | - | - | - | - |  | NT | | NT | NT | | NT | - |
| ΔgG_7284 | - | - | - | - |  | NT | | NT | NT | | NT | - |
| ΔgG_7287 | - | - | - | - |  | NT | | NT | NT | | NT | - |
| ΔgG_7289 | - | - | - | - |  | - | | - | - | | - | + |
| ΔgG_7293 | - | - | - | - |  | - | | - | - | | - | + |
| ΔgG_7295 | - | - | - | - |  | NT | | NT | NT | | NT | - |
| ΔgG_7296 | - | - | - | - |  | NT | | NT | NT | | NT | - |
| ΔgG_7300 | - | - | - | - |  | NT | | NT | NT | | NT | - |
|  |  |  |  |  |  |  | |  |  | |  |  |

^ The nested PCR was applied to swabs only in cases were TG tissue was positive for ILTV DNA

^Ʃ^ NT: not tested with NPCR

^†^ NO: bird lost its identification tag during the trial

^*^ Copies of ILTV DNA per reaction
